# Children’s Individual Differences in Executive Function and Theory of Mind in Relation to Prejudice Toward Social Minorities

**DOI:** 10.3389/fpsyg.2019.02293

**Published:** 2019-10-09

**Authors:** Ángela Hoyo, M. Rosario Rueda, Rosa Rodríguez-Bailón

**Affiliations:** ^1^Mind, Brain and Behavior Research Center, University of Granada, Granada, Spain; ^2^Department of Experimental Psychology, University of Granada, Granada, Spain; ^3^Department of Social Psychology, University of Granada, Granada, Spain

**Keywords:** executive function, theory of mind, prejudice, regulation, cognitive development

## Abstract

Executive function (EF) and theory of mind (ToM) are key cognitive skills for socio-emotional adjustment. Executive function develops considerably between 3 and 7 years of age, and fosters the development of social cognition skills such as ToM. Studies with adults have shown a link between EF and prejudice, as well as between empathy and prejudice. Moreover, the relationship between EF, cognitive and affective ToM and prejudice has barely been studied in children. In this study, we aimed at examining the relationship between individual differences in EF, cognitive and affective ToM and prejudice toward the Romany ethnic minority. We expected a positive association between EF and ToM skills, and a negative association between EF and prejudice. We also predicted a negative association between ToM and prejudice. A total of 86 preschool (5–6 years old, *N* = 43) and third-grade (8–9 years old, *N* = 43) children participated in the study. Results showed a negative relationship between EF and prejudice, as well as between affective ToM and prejudice, after controlling for intelligence. Moreover, we found that EF significantly predicted prejudice. Exploratory correlational analyses suggested age-related differences in the EF skills underlying prejudice regulation. These findings suggest a distinctive contribution of cognitive and affective components of ToM to prejudice, and highlight the central role of EF in social behavior regulation.

## Introduction

In an increasingly globalized world, we live and interact with people coming from diverse ethnic, social and cultural origins. Stereotypes and prejudices toward particular social groups may bias these interactions. In studies with adults, there is evidence that some cognitive skills may help promoting healthy social relationships based on an egalitarian and non-discriminatory behavior (e.g., Bartholow et al., 2006; Lapan and Boseovski, 2015). In this context, abilities necessary to implement goal-directed behavior and understanding others’ thoughts and feelings may strengthen positive interracial interactions. The present study pretends to fill the gap in the existing developmental literature concerning the role played by cognitive skills in the developmental course of prejudice in childhood. The main goals of the current study were: (a) to examine developmental changes in executive function (EF), theory of mind (ToM) and prejudice, and (b) to test relationships between individual differences in EF, ToM, and prejudice in childhood. Moreover, we explored the age-related contributions of those cognitive skills to the expression of prejudice.

### Executive Function, Theory of Mind, and Prejudice: Conceptualization

Different constructs have been proposed to define cognitive skills underlying behavioral regulation, being EF, executive control, and cognitive control examples of them (Diamond, 2013). EF refers to cognitive processes underlying the regulation of thoughts and behavior. EF comprises cognitive flexibility (shifting between rules and mental sets), working memory (WM; updating), and inhibitory control skills (Miyake and Friedman, 2012; Friedman and Miyake, 2017). EFs conform the basis for higher-order cognitive skills contributing to superior functions such as planning, reasoning, and problem-solving (Diamond, 2013) and for what is known as self-regulation (Rueda et al., 2011). A quite established account of executive control considers that two dissociable but intertwined components intervene to implement cognitive control that supports behavior regulation (Bartholow et al., 2006). On the one hand, a conflict detection system, involved in steadily supervision of the ongoing action, detects and signals the need for behavioral adjustment in relation to current goals. On the other hand, a regulatory system directly accounts for behavioral regulation by activating the planned response while inhibiting non-desirable competing responses. Complementarily, it has been shown that, at the neural level, behavior regulation results from two brain networks working with relative independence (Dosenbach et al., 2008; Petersen and Posner, 2012). The cingulo-opercular network is involved in task set maintenance, while the frontoparietal network is engaged in the flexible adjustment of behavior on a trial-by-trial basis. In the present study, we follow Friedman and Miyake’s framework with the aim of disentangling the distinctive contributions of WM, inhibition and cognitive flexibility to behavior regulation in the context of the expression of prejudice.

Concerning ToM, it is a social cognition skill that refers to the ability to attribute mental states to oneself and others (Premack and Woodruff, 1978). It enables people to reason about mental states of other people, as well as to infer the causes of people’s behavior on the basis of the inferred mental states (Wimmer and Perner, 1983). A relatively recent theoretical approach has argued the need of distinguishing between cognitive and affective mental states (Shamay-Tsoory et al., 2010). The relevance of this approach lies in that it has implications for ToM concept and development. Cognitive ToM is the ability to infer people’s beliefs and knowledge. It is also a pre-requisite for affective ToM. Indeed, ascribing an emotion requires a previous understanding of the belief behind that emotion (Miller, 2013). Affective ToM is the ability to infer people’s emotions, and is supported by cognitive and affective empathy. Support for the cognitive-affective ToM division comes from studies finding dissociable brain structures for cognitive and affective empathy (Frith and Frith, 2003; Samson et al., 2004; Dapretto et al., 2006; Schulte-Rüther et al., 2007; Shamay-Tsoory et al., 2009), and for cognitive and affective ToM (Shamay-Tsoory and Aharon-Peretz, 2007). Sebastian et al. (2012) showed evidence that affective ToM is more complex and presents a more protracted development than cognitive ToM, that is, whereas adolescents made more errors in affective ToM tasks than did adults, no differences were found in cognitive ToM performance.

Finally, in intergroup relations, people unfold expectancies in the form of stereotypes about other’s behavior. In fact, stereotypes are often used to judge other people’s actions and they underlie the emergence of negative attitudes toward others because their group membership, that is, the emergence of prejudice (Brown, 2010). Different theories have accounted for the cognitive and environmental factors that underlie the origins and development of prejudice (the *Sociocognitive* approach: Aboud, 1988; the *Social identity Theory* approach: Nesdale and Flesser, 2001; and the *Developmental Intergroup Theory*: Bigler and Liben, 2007). Although they differ each other in the importance given to cognitive and contextual factors, they all claim that prejudice originates as an event linked to the development of skills to group people as a function of social categories.

### Regulating Prejudice Expression: The Role of Executive Function and Theory of Mind

Egalitarian and non-discriminatory behaviors are encouraged and considered socially desirable. Thus, despite most people may be motivated to show prosocial and non-discriminatory behavior during an interracial interaction, they may simultaneously experiment a conflict between their implicit negative beliefs and their motivation to have a non-biased behavior toward people of the outgroup (Amodio, 2014). In this case, people may need to draw on cognitive control as a regulatory mechanism for conflict resolution. Consequently, the engagement of cognitive control in prejudice regulation conveys the EF role in prejudice.

Research on adults has shown that better skills for monitoring the conflict elicited by stereotype-consistent trials (Amodio et al., 2008) and overriding prejudiced impulsive responses (Payne, 2005; Beer et al., 2008) prevent people from expressing automatic bias while performing implicit stereotyping tasks like the Implicit Association Task (IAT; Greenwald et al., 1998). Research manipulating self-regulation demands posed by interracial interaction and including other manipulations that induce cognitive control depletion give additional support to the EF role in prejudice (Richeson and Shelton, 2003; Richeson and Trawalter, 2005; Richeson et al., 2005; Bartholow et al., 2006). Importantly, Bartholow et al. (2006) found that experimenting greater conflict during stereotype-consistent trials in a go-stop stereotype inhibition task was associated with more inhibition errors when stereotype-consistent associations were presented and it was required to withhold the answer. On the contrary, participants that implemented cognitive control in greater extent were better at inhibiting stereotype-consistent answers.

Concerning children, empirical evidence linking EF skills and regulation of prejudice is much scarcer. As a matter of fact, research has mainly focused on the role played by the emergence of early categorization skills in the preschool period in the formation of ingroup and outgroup attitudes (e.g., Aboud, 2008; Enesco et al., 2011; Guerrero et al., 2011). In contrast, significant less research has been devoted to link cognitive skills to reduced racial bias. One instance of these few research is the one by Bigler and Liben (1993). They studied classification skills’ involvement in how Euro-American children aged 4 to 9 years process race-related information. Children’s task was to recall the content of stories that were stereotype-consistent or inconsistent with respect to attributed traits or to the nature of social interactions (intraracial vs. interacial). As predicted, recall for stereotype-inconsistent stories was greater for children that expressed less racial stereotypes and displayed more flexible sorting skills. However, in a more recent study, Patterson and Bigler (2006) found no evidence of classification skills being linked to intergroup attitudes in the preschool period.

Thus, the above-cited literature supports the role of EF in adults’ prejudice regulation, but it is still limited in providing evidence about EF’s involvement in the regulation of prejudice on children samples. Concerning the relationship between ToM and prejudice, research on adults has mostly linked empathy, which is a ToM-related skill, to prejudice. Studies with adults evidence that people improve attitudes toward outgroups if they are encouraged to use their ToM skills and to take the perspective of outgroup members (e.g., Vescio et al., 2003). Pettigrew and Tropp’s (2008) meta-analytical revision confirmed that increased empathy and perspective-taking toward the outgroup mediates the relationship between intergroup contact and decrease of prejudice. In the same line, but with children, better false-belief understanding has been linked to preschoolers’ more positive attitudes toward peers that confront gender stereotypic norms (Mulvey et al., 2016). In a sample of White children belonging to two age groups (6-to-7- and 8-to-9-year-old children), Fitzroy and Rutland (2010) found that higher abilities to perform a second-order false belief task about emotions following a social transgression (Abrams et al., 2009) were related to children’s better control of explicit prejudice irrespective of whether the ingroup norm was for or against prejudice expression. Another ToM-related skill, the so-called self-presentation ToM, is understood as the concern about and regulation of the impression caused on other people, and has been linked to the tendency to make positive trait attributions to outgroup members (Aboud, 2013; Nesdale, 2013; Rutland, 2013). The research by Lapan and Boseovski (2015) is, to our knowledge, the only study that analyzed the role of skills related to both EF and ToM in trait attributions and behavioral predictions held by children 3 to 6 years of age toward typical peers and peers belonging to certain stigmatized social groups (obese children, children with disabilities, and children with foreign accents). They found that only ToM played a role in assessments of characters from stigmatized groups. Better ToM skills were related to more favorable or neutral trait attributions, as well as to more predictions of helping behavior on the part of characters from stigmatized groups.

### Executive Function and Theory of Mind: Development and Developmental Relationships

Different EF components present distinct developmental trajectories. The development of inhibitory control and cognitive flexibility, as well as the contribution of WM development to both abilities, have been studied by Davidson et al. (2006). In their cross-sectional study, different age groups ranging from 4 to 13 years old children and adolescents, as well as a group of 26-year-old young adults were tested on a battery of EF tasks tapping WM, as well as inhibition and cognitive flexibility under different WM demands. In the Dots task, inhibitory control and cognitive flexibility were assessed under high WM demands. In each trial of the Dots task, a single (stripped or gray) dot appeared on the left or on the right side of the screen. Participants’ task was to press as quickly and accurately as possible the same- or opposite-side key to the dot location. Three blocks of 20 trials each were presented. The first two blocks were simple, with one answering rule each. The first simple block required a congruent response, as participants were instructed to press same-side key to the dot location. In the second block, opposite-side (i.e., incongruent) response was required. The third, mixed block, randomly presented 20 congruent and incongruent trials, thus requiring to switch back and forth between congruent and incongruent answering rules. The congruency effect, also called the spatial incompatibility effect (Craft and Simon, 1970), compares performance in congruent and incongruent trials. Thus, it accounts for inhibitory control skills through the cost of inhibiting a dominant response (i.e., press same-side key) when the rule is to press opposite-side key. Cognitive flexibility informs about the differences between simple and mixed blocks, thus comparing performance in single-rule with switching-rule contexts. Results revealed that when inhibitory control is exerted under high memory demands, adults did not show differences in accuracy and reaction time (RT) between congruent and incongruent trials. In children, a reduction of the congruency effect is observed when using accuracy as dependent variable, but not when measured through RT. Regarding cognitive flexibility, a developmental tradeoff tendency between accuracy and RT was found. Accuracy significantly increases from 10 years old children on, as well as RT. This result indicates that, with the development, there is a tradeoff between RT and accuracy in order to preserve a good performance in tasks assessing cognitive flexibility under WM demands.

The findings of Davidson et al. (2006) are in accordance with other studies showing the development of inhibitory control during preschool years (Best and Miller, 2010), as well as further development between early childhood and young adulthood when using fine-grained computerized tasks to assess inhibitory control (e.g., Brocki and Bohlin, 2004; Steinberg et al., 2008). In the same vein, there is evidence of a protracted development of cognitive flexibility, spanning from childhood to early adulthood (e.g., Roberts et al., 1988; Cepeda et al., 2001; Zelazo et al., 2003; Gupta et al., 2009). On the other hand, studies using WM tasks requiring manipulation and updating of information suggest that WM improvement spans from early childhood to middle adolescence (Gathercole et al., 2004; Luciana et al., 2005) and continues until young adulthood (Luciana and Nelson, 1998). Thus, whereas inhibitory control greatly improves in early childhood, WM and cognitive flexibility appear to present a more protracted and linear development (Best and Miller, 2010).

Concerning ToM, developmental studies initially focused on preschoolers’ false-belief understanding, that is, their capacity to understand that people can have wrong beliefs about the world (Miller, 2013). For instance, Wimmer and Perner (1983) found that by 4 years old children started to predict above chance a character’s searching behavior in a transfer task where the character’s belief about the location of an object was wrong. Moreover, by 5 years old, children were able to infer a character’s intention of lying in a situation where different characters have conflicting goals. More recently, Wellman and Liu (2004) informed of a progressive development of understanding the desires, diverse beliefs, false beliefs, beliefs on emotions and real-apparent emotions distinction throughout the preschool period. At age of 3, children consistently start to show understanding of diverse desires and diverse beliefs. Children improve substantially performance on false beliefs when they are 4 years old, and it is at 5 years old when children start to be consistently able to distinguish between real and apparent emotions. However, the understanding of ToM development cannot be constrained to the preschool period, as mastery of false belief understanding does not fully account for ToM development. Other researchers analyzed ToM development beyond the preschool period by using second-order false belief tasks, which test the awareness that someone can hold a false belief about, for instance, another person’s belief. Improvements in the ability to make second-order inferences are observed by 7–8 years old (Perner and Wimmer, 1985; Miller, 2013). It has also been suggested that ToM advances in middle childhood may inform children’s increased flexibility to apply their ToM skills when reasoning about mental states involved in complex social interactions (e.g., Perner, 1988; Miller, 2009; Apperly et al., 2011; Devine et al., 2016). Moreover, conceptual development is probably underpinning ToM improvements as well. In this vein, there is evidence of development of ToM concepts linked with social reasoning and reasoning about ambiguity along middle childhood (Osterhaus et al., 2016). Finally, research using more advanced ToM tests that assess higher orders of recursive thinking has shown further improvements between 14 and 20 years of age (Valle et al., 2015). This result suggests that ToM improvements beyond middle childhood may manifest a more sophisticated use of reasoning skills.

The developmental trajectory of cognitive and affective ToM has also been studied in children. Miller (2013) employed cognitive and affective second-order false belief stories to analyze the development of second-order false belief inference in preschoolers and first-grade children, as well as the effect of content (cognitive vs. affective) on performance. They found a main effect of age: first-grade children outperformed preschoolers when judging the belief of a character about another character’s belief or emotion. Moreover they also found that children found harder to infer second-order beliefs on emotions than on beliefs.

Two main theoretical approaches have been formulated to account for the developmental relationship between EF and ToM. The *emergence* account highlights the EF role in the rise and development of ToM (e.g., Russell, 1996), and the ToM role in EF development (e.g., Perner and Lang, 1999, 2000). The *expression* account stresses that EF is involved in ToM in the extent to which performance in ToM tasks demands the use of cognitive control skills just in order to unfold ToM knowledge (e.g., Perner et al., 2002b). Therefore, overall, existing research supports the role of EF in the emergence of ToM.

In preschoolers, earlier EF predicts later ToM performance across cultures and after controlling for age and verbal ability (Devine and Hughes, 2014). In middle childhood, whereas Devine et al. (2016) did not find longitudinal association between EF and ToM, Lecce et al. (2017) showed that early WM predicted later ToM performance in a longitudinal study following children aged 9.5 to 10.5 years old, providing further support for the emergence account.

### Origins and Development of Prejudice in Childhood

Different approaches (e.g., Aboud, 1988; Nesdale and Flesser, 2001; Bigler and Liben, 2007) claim that the emergence of prejudice is linked to the development of categorization, a skill necessary to group people as a function of social categories. As children are able to distinguish between the ingroup and differentiate it from the outgroups, they tend to display ingroup favoritism and outgroup rejection, especially toward minority groups. Concerning developmental changes in prejudice, there is evidence indicating some differences between the developmental course of Spanish children and children who grow up in societies that more ethnically diverse for longer time. The first studies carried out in the 90’s showed that whereas children from traditional multi-ethnic societies unfold abilities to categorize people on the basis of race when they are 3–4 years old (Holmes, 1995), it is only at age 7 when Spanish children consistently show ability to classify people according to the skin color (Enesco et al., 1999). Studies that account for changes in prejudice and have been carried out in traditionally multi-ethnic countries have found a significant counter-bias increase between preschoolers and third-graders, as well as no changes in prejudice (e.g., Doyle and Aboud, 1995). Raabe and Beelmann (2011) meta-analytical revision documented a peak in explicit racial prejudice between 5 and 7 years old, followed by a significant decrease in late childhood, between 8 and 10 years old. In the Spanish context lately the Spanish population has become more and more multi-ethnic due to immigration. This fact is likely to have impacted on the developmental course of prejudice. In fact, more recently it has been found that Spanish children show the peak of prejudice at around age 6, similarly to children coming from multiethnic societies (Enesco et al., 2008). However, it has also been suggested that Spanish children’s developmental decreases in prejudice may still not be evident until early adolescence (Enesco et al., 2005).

### The Present Study

As it has been shown, the vast majority of research on the relationship between EF and prejudice has focused on adult samples. Empirical evidence about the distinctive contribution of inhibitory control, cognitive flexibility, and WM skills to regulation of prejudice is needed. In fact, it is necessary to elucidate whether developmental decrement in prejudice is due in greater extent to age-related improvement in cognitive flexibility skills, that enable children to question stereotypes and limit their use, or to older children’s ability to inhibit their stereotypes and give socially desirable answers instead (Enesco et al., 2005). In line with what Enesco et al. (2009) suggest, there is a chance that the age-related enhancement of cognitive flexibility skills allows a more refined social information processing, and therefore is likely to play a role in regulation of prejudice. Hence, additional research with children samples is needed. Additionally, research linking ToM and prejudice is still scarce in children. Research on prejudice with both adults and children (e.g., Nesdale et al., 2005; Pettigrew and Tropp, 2008) has given a central role to empathy. To our knowledge, only Fitzroy and Rutland (2010) investigated children’s affective mental state understanding in connection with the expression of prejudice. Therefore, the distinctive contribution of cognitive versus affective ToM components to prejudice remains unknown. In the present study, we assessed preschool and third-grade children. The election of these age groups was done on the basis of empirical evidence showing that developmental changes in EF and ToM are expected between early and middle childhood, Moreover, according to previous studies on children’s categorization skills and stereotypic attributions using Spanish samples, we considered that developmental changes in prejudice may presumably take place between early and middle childhood. Therefore, the first goal of the present research was to explore developmental changes in EF, ToM and prejudice, while controlling by intelligence. We expected age-related improvements in all cognitive skills. According to previous studies (e.g., Raabe and Beelmann, 2011), we also expected to find an age-related decrease in explicit prejudice. Secondly, we examined the relationship between EF and ToM, and whether individual differences in EF and ToM significantly relate to prejudice. We aimed at exploring distinctive contributions of inhibitory control, cognitive flexibility and WM, as well as of cognitive and affective ToM. In light of previous findings (e.g., Amodio et al., 2008; Fitzroy and Rutland, 2010; Carlson et al., 2015), we expected a positive relation between EF and ToM, a negative relation between EF and prejudice, and a negative relation between ToM and prejudice. Finally, we explored age-related differences in the contribution of cognitive skills to the expression of prejudice.

## Materials and Methods

### Participants

A total of 86 children, divided into two age groups (preschool and third-grade children), participated in the study. Preschool children aged 5 to 6 years (*N* = 43, mean age = 69.86 months, *SD* = 4; 21 girls), and third-grade children aged 8 to 9 years (*N* = 43, mean age = 107.54 months, *SD* = 4.22; 25 girls), participated in the study. Two third-grade children presented data missing in the IQ score. Children were recruited from two schools located in middle socioeconomic status districts of Granada (Spain). They were all Caucasian and did not have learning difficulties or history of psychological disorders.

### Procedure

The study obtained approval from the University of Granada Ethics Committee. All participants had informed parental consent. Participants were assessed in two different sessions, each one lasting 30 min approximately. Participants performed intelligence and EF tasks in the first session, and ToM and prejudice tasks in the second. The assessment sessions were carried out individually in a separated and quiet room of the school.

### Measures

#### Intelligence

We used the Spanish adaptation of the K-BIT (Kaufman Brief Intelligence Test; Cordero and Calonge, 2009). This test provides vocabulary, matrices, and the composite intelligence (IQ) scores. Vocabulary is a measure of crystallized intelligence, whereas matrices is a measure of fluid intelligence. The composite IQ score was included as a control variable in correlational analyses.

#### Executive Function

**FIGURE 1:**
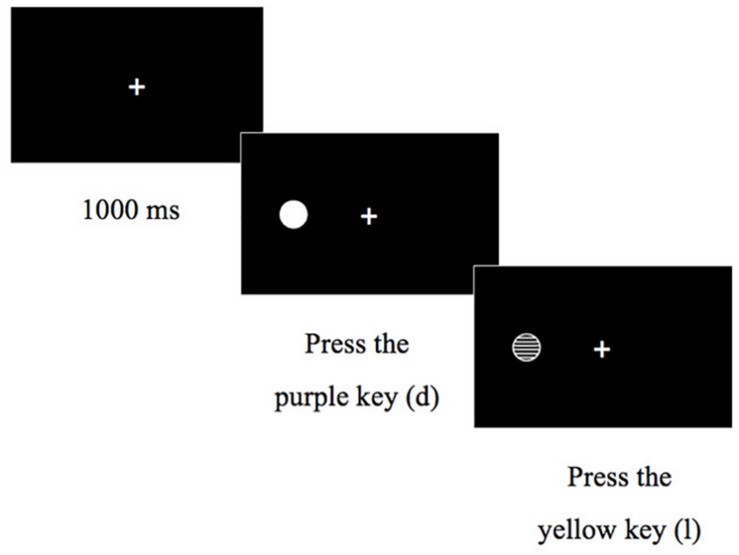
DOTS task.

A new version of the Dots spatial conflict task (Davidson et al., 2006) was used to measure conflict resolution and cognitive flexibility EF components (see Figure 1). Our Dots task differed from that of Davidson et al. (2006) as follows: (a) the stimulus-response mapping was the same for all participants; (b) blocks of trials where presented in a fixed order. First, two simple blocks of 24 trials each were presented, the first with congruent trials and the second with incongruent trials, followed by two mixed blocks of 24 trials each, containing half congruent and half incongruent trials randomly selected; (c) instructions and practice were provided at the beginning of the task and before starting the mixed blocks; and (d) we established the same trial duration for both age groups. Each trial started with a fixation point (1000 ms) on the center of the screen. Next, the stimulus appeared randomly on the left or on the right side of the screen during 2500 ms. Stimuli were white dots and dots with horizontal white and black stripes. In congruent trials, children were instructed to press as quickly and accurately as possible the key in the same side of the white dot, whereas in incongruent trials, children had to press the key in the opposite side to the striped dot. Children had to press one of two possible keys (d or l), identified with stickers. Children performed three blocks of trials in a fixed order. Firstly, a congruent block required pressing the key that matched the stimulus position. Next, an incongruent block asked children to press the opposite key to the stimulus position. Finally, the mixed block combined random congruent and incongruent trials. There were breaks between blocks, and one break after half of the trials in the mixed block. Duration of breaks was flexible and long enough to let children rest but also keep their engagement in the task. Congruent and incongruent were simple, non-switch blocks, with one answering rule each. The mixed block was a switch block requiring children to flexibly select the appropriate response according to the dot pattern presented in each trial. The response rule was remembered at the beginning of each block. Each block had four practice trials. Simple (congruent and incongruent) blocks had 24 trials each, and the mixed block had 48 trials. We calculated three scores on the basis of participants’ RT:

Simple⁢conflict⁢resolution=

Incongruent⁢Block⁢Median⁢RT-Congruent⁢Block⁢Median⁢RT

Mixed⁢conflict⁢resolution=Mixed⁢Incongruent⁢Trials⁢Median

RT-Mixed⁢Congruent⁢Trials⁢Median⁢RT

Cognitive⁢flexibility=Median⁢Mixed⁢Block-Mean

(Incongruent⁢Block⁢Median⁢RT+Congruent⁢Block⁢Median⁢RT)

Conflict resolution scores are a measure of children’s ability to inhibit a prepotent response (press same-side key) in favor of non-automatized, goal-directed behavior (press the opposite-side key in incongruent trials). Thus, conflict resolution indexes children’s ability to deal with the spatial incompatibility effect (Craft and Simon, 1970), with greater scores indicating less conflict resolution skills. Simple conflict resolution (without flexibility load) accounts for the child’s ability to overcome the automatic tendency to press same-side key by comparing single-task blocks (that is to say, two blocks with one answering rule each and, hence, one task set each), so no flexibility demands are posed. Mixed conflict resolution (with flexibility load) tests performance under flexibility demands. Then, it accounts for the size of the spatial incompatibility effect in a set-switching context, i.e., the context of the mixed block requiring to sometimes switching between two task sets. Finally, the cognitive flexibility score is an index of the task switching cost. It compares performance in contexts that require task set maintenance (i.e., the single-rule, simple blocks) with performance in a context that, on a trial-by-trial basis, demands the flexible selection and use of two task rules (i.e., the mixed block). Then, greater cognitive flexibility scores indicate more task switching costs. To ease interpretation in correlational analysis, conflict resolution and cognitive flexibility scores were reversed to indicate better EF skills.

#### Working Memory

We assessed WM with the digit span task from the Wechsler Intelligence Scale for Children (WISC-IV; Corral et al., 2005). Children first listened to series of numbers, and then repeated them aloud in direct (forward) and reverse (backward) order. There were eight elements in each order. Children were presented two series per element. Series gradually increased in length. The test finished if the child failed the two series of a particular length. Children received one point for each correctly repeated series. WM score was the sum of correctly repeated backward series.

#### Cognitive Theory of Mind

Cognitive ToM tasks consisted of deceptive container tasks, a first-order false belief task (Sally-Anne task from Baron-Cohen et al., 1985), and cognitive second-order false belief stories from Miller (2013).

For the deceptive container tasks, a piggy bank with marbles and a pencil case with candles were used. Children saw the container and were asked about the expected content. Then, the experimenter showed the real content and saved it again. Now the experimenter asked what a new child would think that there was inside. Children received one point if the comprehension and the ToM questions in each task were correctly answered. Score ranged from 0 to 2.

In our Sally-Anne task, the characters were Silvia and Ana. Silvia puts a red ball inside a basket. Then, she goes out, and in the meantime Ana puts the ball in a box. Then, Ana goes out and, after a while, Silvia comes back. Children were asked comprehension (“Where is the ball hidden?”) and ToM questions (“Where does Silvia think that the ball is hidden?”). Children received one point if they correctly answered both questions. Score ranged from 0 to 1.

Cognitive second-order beliefs are beliefs about others’ thoughts. We made use of two cognitive second-order false belief stories from Miller 2013; see Supplementary Material). With the help of vignettes, the experimenter read aloud the stories. Next, children were asked two comprehension questions and two ToM questions. The first ToM question requires a judgment of a character’s belief about the thought of another character (e.g., “Where does Ana think Juan has gone?”). In case the child did not answer that question, judgment was assessed with a forced choice question (e.g., “Does Ana think Juan has gone to the soccer field or to the park?”). The second ToM question targets at the justification of that character’s belief (e.g., “Why does Ana think Juan is there?”). Two points were awarded if both judgment and justification questions were correctly answered, one point if children only failed the justification, and zero if they failed both ToM questions.

#### Affective Theory of Mind

To assess affective ToM, we designed a hidden emotion task to examine real and apparent emotion distinction, and also included the affective second-order false belief stories from Miller (2013).

In the hidden emotion task, the experimenter presented the story by using vignettes. In the story, Pablo is going to celebrate his birthday next Friday. In the way to school, he sees several toys in a toys shop’s window, and thinks of the toy he would like to receive (a racing car). Finally, his grandmother gifts him a fluffy toy. Pablo smiles and thanks his grandmother. The comprehension questions were: “Which gift did Pablo want?” and “Which gift did Pablo receive?” Then the experimenter introduced Pablo’s real emotion ToM question: “When Pablo receives the fluffy toy, he smiles. How do you think that Pablo feels?” Afterward, children rated Pablo’s emotion according to a scale of faces (see Supplementary Material). The scale consisted in seven schematic faces depicting subtle changes in facial expressions depicting sad (faces 1–3), neutral (face 4), and happy (faces 5–7) emotions. If children had correctly answered the ToM question and had chosen a face that properly identified Pablo’s real emotion (that is, sadness), the experimenter asked: “If Pablo does not feel well, why do you think that he smiles?” Children received one point for each correct answer (ToM question, scale of faces, and justification), so scores ranged from 0 to 3.

Beliefs about others’ emotions were assessed with two affective second-order false belief stories (Miller, 2013; see Supplementary Material). Similarly to the cognitive stories, after reading each story children were asked two comprehension questions and two ToM questions. Now the first ToM question requires a judgment of a character’s belief about the emotion of another character (e.g., “How does Antonio think María is feeling before he finds her?”), and in case of no answer, the forced choice question was made (e.g., “Does Antonio think María is feeling happy or sad?”). In the justification question, children were asked to give reasons for that character’s belief (e.g., “Why does Antonio think that María is feeling that way?”). Two points were awarded when children gave correct answers to judgment and justification questions. Children received one point if they only failed the justification. Zero points were given to children failing both ToM questions.

#### Prejudice

In order to assess prejudice we used the Multi-response Racial Attitude task (MRA; Doyle and Aboud, 1995). We employed drawings depicting ingroup (Caucasian) and outgroup (Romany) children members. We chose the Romany outgroup given that the Romany group is one of the most prominent minority groups in Spain (Enesco et al., 2005). Twenty adjectives and behavioral descriptions for each one were presented. Ten adjectives described positive traits (clean, wonderful, healthy, good, nice, happy, friendly, kind, helpful, and smart), and the other 10 described negative traits (unfriendly, mean, dirty, cruel, stupid, selfish, sick, naughty, sad, and bad). The adjectives, along with behavioral descriptions, were read aloud to children. After reading each adjective, children were asked to point at the child they thought that could be or behave in that way. Children could point at one, both, or any of the drawings. This procedure ensured children did not make a forced assignation of traits. We obtained ingroup and outgroup attitude scores. They were calculated by subtracting negative from positive assigned traits. Thus, the resulting score would range from −10 to 10. Negative scores indicated more attribution of negative than positive traits, whereas positive scores indicated more attribution of positive than negative traits. A composite prejudice score was calculated by subtracting outgroup from ingroup attitudes. This composite score ranged from −20 to 20. Positive scores informed more negative attitudes toward the outgroup than to the ingroup. In line with Doyle and Aboud (1995), we also calculated a counter-bias score by summing positive outgroup and negative ingroup assigned traits.

## Results

All third-grade children correctly answered to the deceptive container task, so this score was removed from the analyses. Correlation between Silvia-Ana and cognitive second-order false belief was moderate (*r* = 0.34, *p* = 0.001). In order to obtain a total cognitive ToM score, we calculated the mean of the *z* scores for each task.

Correlation between affective ToM tasks was moderate (*r* = 0.40, *p* < 0.001). A total affective ToM score was obtained by calculating then mean of the *z* scores for each task. Means and standard deviations of the raw scores in ToM tasks and in the Dots task for each age group are shown in Tables 1, 2, respectively.

**TABLE 1:** Means and (standard deviations).

**Age group**	**Content**				
	**Cognitive**			**Affective**	
	**Deceptive container**	**Silvia-Ana**	**Cognitive ToM second-order**	**Real-apparent emotion**	**Affective ToM second-order**
	***M (SD)***	***M (SD)***	***M (SD)***	***M (SD)***	***M (SD)***
5–6	1.44 (0.83)	0.70 (0.46)	1.23 (0.94)	0.81 (1.16)	1.19 (1.12)
8–9	2.00 (0)	0.95 (0.21)	2.72 (1.22)	1.65 (1.04)	2.35 (1.23)

*ToM tasks.*

**TABLE 2:** Means and (standard deviations).

**Age group**	**Condition**							
	**Simple**				**Mixed**			
	**Congruent**		**Incongruent**		**Congruent**		**Incongruent**	
	**Reaction time**	**Errors (%)**	**Reaction time**	**Errors (%)**	**Reaction time**	**Errors (%)**	**Reaction time**	**Errors (%)**
	***M (SD)***	***M (SD)***	***M (SD)***	***M (SD)***	***M (SD)***	***M (SD)***	***M (SD)***	***M (SD)***
5–6	671.92 (149.69)	2.71 (4.71)	969.58 (228.74)	9.30 (7.87)	1178.47 (210.63)	11.43 (10.95)	1206.64 (216.73)	14.24 (12.44)
8–9	529.00 (163.29)	0.97 (2.00)	703.02 (209.62)	5.43 (6.90)	908.15 (220.62)	9.50 (10.62)	938.05 (245.36)	5.23 (6.31)

*Dots task.*

**FIGURE 2:**
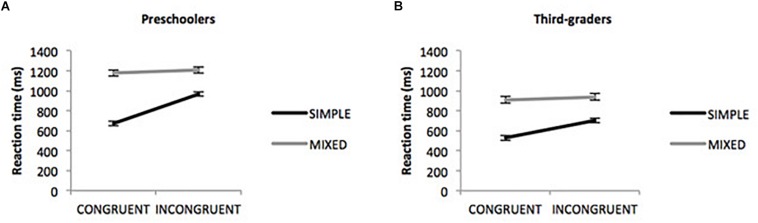
**(A)** Preschoolers’ reaction time in DOTS task as a function of Block, Congruency, and Age Group. **(B)** Third-graders’ reaction time in DOTS task as a function of Block, Congruency, and Age Group.

Regarding the Dots task, we ran a repeated measures ANOVA on RT, with Congruency (congruent vs. incongruent) and Block (simple vs. mixed) as within-subject factors and Age group as the between-subject factor. Anticipatory responses (>200 ms) and errors were filtered out. For greater clarity, only relevant results for our purposes will be reported. All main effects were significant [Congruency (*F*(1,84) = 188.08, *p* < 0.001, ${}_{\text{p}}^{2}$ = 0.69); Block (*F*(1,84) = 517.40, *p* < 0.001, ${}_{\text{p}}^{2}$ = 0.86); Age group (*F*(1,84) = 35.09, *p* < 0.001, ${}_{\text{p}}^{2}$ = 0.30). Participants took more time to respond to incongruent than congruent trials (*d* = 132.44, *p* < 0.001), and in the mixed block compared to the simple blocks (*d* = 339.45, *p* < 0.001). In addition preschoolers showed longer RTs than third-graders (*d* = 237.10, *p* < 0.001). The three-way interaction of Congruency × Block × Age group was also significant, *F*(1,84) = 10.79, *p* < 0.01, ${}_{\text{p}}^{2}$ = 0.11. To breakdown this three-way interaction, two repeated measures ANOVAs were performed with each age group (see Figure 2). For preschoolers the interaction between Congruency and Block was significant, *F*(1,42) = 63.66, *p* < 0.001,${}_{\text{p}}^{2}$ = 0.60. The difference in RT between incongruent and congruent trials was significant in the simple blocks (*d* = 297.66, *p* < 0.001) but not in the mixed one (*d* = 28.17, *p* = 0.26). For third-graders the interaction between Congruency and Block was also significant, *F*(1,42) = 65.68, *p* < 0.001, ${}_{\text{p}}^{2}$ = 0.61. The difference in RT between incongruent and congruent trials was significant in the simple blocks (*d* = 174.04, *p* < 0.001) and also in the mixed one (*d* = 29.91, *p* < 0.05). Consequently, significant main and interaction effects of Congruency, Block and Age group make feasible to include conflict resolution and cognitive flexibility scores in analyses aimed at testing our hypotheses. For that purpose we calculated the scores mentioned in section “Materials and Methods.”.

### Developmental Changes in Cognitive Skills

In order to analyze age-related changes in the studied cognitive skills, we performed *t*-tests for independent samples. As it was expected, third-graders outperformed preschoolers in EF and ToM measures. However, contrary to expected, no age-related differences in prejudice were found (see Table 3). The *post hoc* statistical analyses revealed an achieved power of 1 for each of the following scores: simple conflict resolution, WM, cognitive ToM and affective ToM. For mixed conflict resolution, the difference was not significant (achieved power = 0.05). For the cognitive flexibility score, the difference was marginal (*p* = 0.053; achieved power = 0.49). Contrary to what we expected, no significant age-related decrease in prejudice and increase in counter-bias were found (achieved power = 0.07 and 0.26 respectively). We also calculated the effect size of the differences between age group means using Cohen’s *d*. Regarding EF, small effect sizes were found in the DOTS task (simple conflict resolution = 0.01; mixed conflict resolution = 0.02; cognitive flexibility = 0.42), and a large effect size on the WM score, *d* = 1.57. For differences between age groups on ToM performance, all effect sizes were large (cognitive ToM = 1.66; affective ToM = 1.09). Small effect sizes were found for the prejudice scores (prejudice = 0.1; counter-bias = 0.29). Finally, there were significant differences between age groups in matrices and composite IQ scores. We accounted for age-related IQ differences by including the composite IQ as a control variable in subsequent analyses.

**TABLE 3:** Results of *t*-test comparing age groups in all measures.

		**Group**							
		**Preschoolers**	**Third-graders**	**Mean difference**	**Standard error difference**	**95% CI for mean difference**	**Levene’s test**		
							
		***M* (*SD*)**	***M* (*SD*)**				***F***	***t*^1^**	***df*^1^**
Intelligence	Vocabulary	108.05(11.67)	107.07(10.82)	0.98	2.33	−3.65, 5.60	0.02	0.42	84
	Matrices	112.28(12.06)	98.44(20.31)	13.84	3.62	6.63, 21.05	2.24	3.82^∗∗∗^	82
	Composite IQ	109.23(9.72)	102.17(12.90)	7.06	2.49	2.12, 12.01	3.57#	2.84^∗∗^	82
Ejecutive function	Simple conflict resolution	285.65(127.68)	174.03(98.90)	111.61	24.63	62.64, 160.59	2.62	4.53^∗∗∗^	84
	Mixed conflict resolution	28.17(160.91)	29.91(87.11)	–1.74	27.90	−57.47, 53.99	11.80^∗∗∗^	–0.06	65
	Cognitive flexibility	362.64(141.32)	303.62(137.62)	59.02	30.08	−0.80, 118.85	0.12	1.96#	84
	Working memory	4.60(0.90)	6.26(1.20)	–1.65	0.23	−2.11, −1.20	2.79#	–7.22^∗∗∗^	84
Theory of mind	Cognitive ToM	−0.45(0.77)	0.45(0.59)	–0.90	0.15	−1.19, −0.60	4.70^∗^	–6.07^∗∗∗^	84
	Affective ToM	−0.40(0.74)	0.40(0.73)	–0.80	0.16	−1.12, −0.48	0.26	–5.03^∗∗∗^	84
Prejudice	Prejudice	9.84(7.09)	10.49(6.15)	–0.65	1.43	−3.50, 2.19	0.54	–0.46	84
	Counter-bias	6.98(4.93)	8.53(5.88)	–1.56	1.17	−3.89, 0.077	2.27	–1.33	84

*^∗^*p* < 0.05; ^∗∗^*p* < 0.01; ^∗∗∗^*p* < 0.001; ^#^*p* > 0.05.^1^The reported Student’s *t* and degrees of freedom when the Levene’s test is significant is the corrected t. Cognitive ToM, cognitive theory of mind; Affective ToM, affective theory of mind.*

### Individual Differences in Executive Function and Theory of Mind and Its Relation With Prejudice

Firstly, we carried out one-tailed partial correlations controlled by composite IQ (see Table 4) with the whole sample, in order to analyze the relationship between individual differences in the cognitive skills of interest (that is, EF and ToM) and prejudice. Moreover we included participants’ age (in months) for further testing the role of age in EF, ToM, and prejudice. The composite IQ was added as a control variable given that age group differences in IQ were observed. Literature has consistently reported a link between EF and IQ (e.g., Arffa, 2007). Moreover, when studying the contribution of EF to ToM, researchers (e.g., Carlson et al., 2015; Lecce and Bianco, 2018) usually make use of verbal ability tasks given the overlap between verbal intelligence and ToM. Accordingly, we controlled by IQ in order to ensure that the relationships between cognitive skills and prejudice are due to individual differences in such cognitive skills and not due to age group differences in IQ. As noted in the Method, EF scores were reversed, with higher scores indicating better EF skills. Results showed expected significant associations of age with EF and ToM, and pointed toward the expected relationship between age and prejudice. Age was positively correlated with simple conflict resolution, cognitive flexibility and WM. *Post hoc* statistical analyses revealed an achieved power of 1 for correlations of age with simple conflict resolution and WM, and of 0.58 for the correlation between age and cognitive flexibility. There was a strong relationship between age and both cognitive and affective ToM (achieved power = 1 for both). Concerning prejudice, a marginal positive relationship between age and counter-bias was found (achieved power = 0.48). As expected, results showed a significant relationship between EF and ToM. None of the correlations between mixed conflict resolution and the other variables were significant. In contrast, simple conflict resolution positively correlated with both cognitive and affective ToM. Cognitive flexibility positively correlated with affective ToM and tended to be positively correlated with cognitive ToM (*p* = 0.07). Moreover, WM was positively associated with both cognitive and affective ToM. Results also confirmed our prediction regarding the link between EF and prejudice. Indeed, there was a significant negative relationship between EF and prejudice. Correlations showed that the less cognitive flexibility the more prejudice, and in the same line, a positive relation between cognitive flexibility and the measure of counter-bias. Moreover, WM was positively associated with counter-bias. The *post hoc* statistical analysis revealed an achieved *post hoc* power of 0.84 for the correlation between cognitive flexibility and prejudice, and an achieved power of 0.62 for the correlation between WM and counter-bias. Finally, only affective ToM was positively associated with counter-bias (achieved power = 0.62).

**TABLE 4:** One-tailed correlations controlled by total IQ.

	**1**	**2**	**3**	**4**	**5**	**6**	**7**	**8**
(1) Age (months)	–							
(2) Simple conflict resolution	0.51^∗∗∗^	–						
(3) Mixed conflict resolution	0.06	0.07	–					
(4) Cognitive flexibility	0.19^∗^	0.15#	0.12	–				
(5) Working Memory	0.60^∗∗∗^	0.33^∗∗∗^	0.11	0.26^∗∗^	–			
(6) Cognitive ToM	0.55^∗∗∗^	0.21^∗^	0.13	0.17#	0.42^∗∗∗^	–		
(7) Affective ToM	0.49^∗∗∗^	0.19^∗^	–0.04	0.29^∗∗^	0.42^∗∗∗^	0.57^∗∗∗^	–	
(8) Prejudice	0.03	–0.03	–0.02	–0.28^∗∗^	–0.09	0.10	–0.13	–
(9) Counter-bias	0.16#	0.14	0.01	0.26^∗∗^	0.21^∗^	0.03	0.21^∗^	–0.92^∗∗∗^

**^∗^p* < 0.05; *^∗∗^p* < 0.01; *^∗∗∗^p* < 0.001; #*p* > 0.05. Cognitive ToM, cognitive theory of mind; Affective ToM, affective theory of mind.*

Next, in light of the significant correlations found, we performed stepwise regression analyses. We included IQ (and age if necessary) in the first step, EF in the second step and ToM in the third step. First we used the composite IQ and cognitive flexibility scores as predictors of prejudice. The model was significant (Δ*R*^2^ = 0.08, *p* < 0.05; *F* = 7.04, *p* = 0.01), and cognitive flexibility was the only significant predictor (β = −0.28, *p* = 0.01). Secondly, in order to predict the counter-bias score, we used the age in moths, composite IQ, cognitive flexibility, WM and affective ToM as predictors. The model was significant (Δ*R*^2^ = 0.07, *p* < 0.05; *F* = 6.05, *p* < 0.05). Cognitive flexibility was the only significant predictor included in the model (β = 0.26, *p* < 0.05).

### Exploratory Analyses on Age-Related Distinctive Contributions of Cognitive Skills to Prejudice

As noted in the goals of the study, we were interested in exploring possible distinctive age-related contributions of EF and ToM to prejudice. Consequently, we performed one-tailed correlations for the two age groups (see Table 5). For preschoolers the expected relationship between EF and ToM was just marginally significant between simple conflict resolution and cognitive ToM (*p* = 0.08; achieved power = 0.43). Interestingly, a significant relationship between EF and prejudice emerged. Both cognitive flexibility and WM negatively correlated with prejudice (achieved power = 0.69 for both relationships) and positively with counter-bias (achieved power = 0.49 for cognitive flexibility and 0.69 for WM). Finally, a marginal relationship in the expected direction between affective ToM and prejudice was found (*p* = 0.08; achieved power = 0.43). Concerning third-graders, the analyses showed significant associations between EF and ToM, and importantly, between EF and prejudice. Specifically, cognitive flexibility was positively associated with affective ToM (achieved power = 0.52). Moreover, both simple conflict resolution and cognitive flexibility were negatively associated with prejudice and positively associated with counter-bias. For the simple conflict resolution, achieved power was 0.57 for the association with prejudice and 0.68 for the association with counter-bias. Achieved powers of 0.63 and 0.58 were respectively found for the relation between cognitive flexibility and prejudice and between cognitive flexibility and counter-bias. Finally, we performed stepwise regression analyses for each age group. Following the same analytic strategy used for the previous analysis for the whole sample, only variables that had significantly correlated were considered. We introduced IQ in the first step, EF in the second step, and ToM in the third one. For preschoolers, regression analysis to predict prejudice included IQ, cognitive flexibility, WM, and affective ToM. The model was significant (Δ*R*^2^ = 0.10, *p* < 0.05; *F* = 4.55, *p* < 0.05). Cognitive flexibility was the only significant predictor included in the model (β = −0.32, *p* < 0.05). The model for predicting counter-bias included IQ, cognitive flexibility and WM. In this case, none of the predictors reached significance. For third-graders, a first regression analysis with prejudice as dependent variable and IQ, simple conflict resolution and cognitive flexibility as predictors was not significant for any of the variables. In contrast, the regression analysis predicting counter-bias from IQ, simple conflict resolution and cognitive flexibility resulted in a significant model (Δ*R*^2^ = 0.11, *p* < 0.05; *F* = 4.56, *p* < 0.05), being simple conflict resolution the significant predictor (β = 0.32, *p* < 0.05).

**TABLE 5:** Correlations split by age group.

	**Age group**	**1**	**2**	**3**	**4**	**5**	**6**	**7**
(1) Simple conflict resolution	5–6	–						
	8–9	–						
(2) Mixed conflict resolution	5–6	–0.01	–					
	8–9	0.20						
(3) Cognitive flexibility	5–6	0.09	0.16	–				
	8–9	0.31^∗^	0.04					
(4) Working memory	5–6	0.04	0.05	0.31^∗^	–			
	8–9	0.06	0.21#	0.14				
(5) Cognitive ToM	5–6	0.23#	0.13	0.06	0.18	–		
	8–9	–0.19	0.16	0.14	–0.05			
(6) Affective ToM	5–6	0.09	–0.09	0.20	0.15	0.33^∗^	–	
	8–9	–0.01	0.06	0.26#	0.15	0.51^∗∗∗^		
(7) Prejudice	5–6	0.08	0.04	−0.32^∗^	−0.32^∗^	0.13	−0.22#	–
	8–9	−0.29^∗^	–0.16	−0.30^∗^	–0.04	0.02	–0.16	
(8) Counter-bias	5–6	0.12	0.08	0.24#	0.32^∗^	–0.13	0.17	–0.92^∗∗∗^
	8–9	0.31^∗^	0.15	0.28^∗^	0.05	–0.01	0.20	–0.98^∗∗∗^

*One-tailed correlations controlled by total IQ. *^∗^p* < 0.05; *^∗∗∗^p* < 0.001; ^#^*p* > 0.05. Cognitive ToM, cognitive theory of mind; Affective ToM, affective theory of mind.*

## Discussion

The first goal of the present research was to analyze age-related changes in EF, ToM, and prejudice in children aged from 4 to 9. Secondly, we examined whether individual differences in EF and ToM are associated with prejudice. Finally, we explored age-related differences in the contribution of cognitive skills to the expression of prejudice. As noted above, previous research mainly using adult samples has shown a link between EF and prejudice (e.g., Bartholow et al., 2006), as well as between empathy and prejudice (e.g., Pettigrew and Tropp, 2008). The present research aimed at gaining a better understanding of the role of cognitive skills in the expression of prejudice in children.

Firstly, results showed an overall age-related improvement in the Dots task. Importantly, the manipulations had the expected effect. Indeed, participants responded faster to congruent than incongruent trials, as was expected according to the spatial incompatibility effect (Craft and Simon, 1970). Interestingly, a three-way interaction between Congruency, Block and Age group was found. In the mixed block, whereas third-grade children still showed the spatial incompatibility effect (though it was low), preschoolers found congruent and incongruent trials equally difficult. Arguably, this result suggests that older children are better able to maintain the task set and adjust responses accordingly. Conversely, younger’s children executive resources seem to be exhausted by maintaining and switching between rules, thus congruent and incongruent trials become equally difficult. This is reflected in larger overall RT in the mixed block and in the lack of congruency effect observed only in this block and only for young children. The so-called global context effect (Davidson et al., 2006) claims that performance is affected not only by the nature of a single trial, but also by the context in which the trial is presented. In the mixed block, the changing rule overloads the requirement of executive control, which affect the adjustment of responses in both congruent and incongruent trials. As expected, the switching context of the mixed block influenced children’s performance.

### Developmental Changes

With respect to results on developmental changes, a global age-related improvement on performance was found. Concerning EF, both inhibitory control and WM significantly improve between early and middle childhood. This result is consistent with previous research that accounts for inhibitory control improvements beyond early childhood (e.g., Brocki and Bohlin, 2004; Steinberg et al., 2008) and with research suggesting a protracted WM development (Luciana and Nelson, 1998; Gathercole et al., 2004). For cognitive flexibility, only marginally significant age-related gains were found. We argue that there is a chance that the allowed response time (2500 ms) has affected the task’s sensitivity to capture slight but significant developmental changes. In a previous research using the Dots task (Davidson et al., 2006), the allowed response time was 2500 ms for children between 4 and 6 years old and 1250 ms for participants aged 7 and older, as well as for a sub-group of 6-year-olds. It is likely that our Dots task posed less cognitive demands than the Dots task used by Davidson et al. (2006), that is, older children in our study might have taken advantage of the 2500 ms allowed response time and accordingly the difficulty of the task diminished as children had more time to respond. In fact, in our study, 8-to-9-year-old children had a mean RT in the mixed block of around 200 ms larger than in Davidson et al. (2006) study. Consequently, the task may not have precisely detected older children’s individual differences in cognitive flexibility skills. With regard to ToM, our results expand previous findings (Miller, 2013) by showing that the development of cognitive and affective ToM goes beyond the early school years and continue along middle childhood. Finally, contrary to what we expected, results showed no age-related changes in prejudice. In relation to it, we found neither a significant age-related prejudice decrease nor a significant counter-bias increase. Previous studies mostly based on research in traditional multi-ethnic Western countries have documented a decrease in explicit racial prejudice (Raabe and Beelmann, 2011), as well as a counter-bias increase (Doyle and Aboud, 1995) in late childhood, from age 8 on. In the Spanish context, studies on the development of prejudice along childhood, though still scarce, have provided valuable evidence about singularities of Spanish children’s development of prejudice. For instance, whereas children from traditional multi-ethnic societies categorize people on the basis of race at 3–4 years old (Holmes, 1995), Enesco et al. (1999) found that Spanish children were not consistently able to use skin color as a classification criterion until 7 years old. However, more recently it has been suggested that Spanish children categorize people according to skin color earlier in the development, at around age 6 (Enesco et al., 2008). Enesco et al. (2005) found that children’s agreement with stereotypes held by society toward Romany people decrease between second and sixth grades (7–11 years old). Then, it could be possible that the age range considered in our study is not wide enough to capture significant developmental changes linked to prejudice regulation.

### EF and ToM in Relation With Prejudice: Individual Differences and Age-Related Distinctive Contributions

Finally, we were interested on studying the relationships between individual differences in EF, ToM and prejudice. We also explored possible age-related differences in the contribution made by EF and ToM to prejudice. As expected, and according to previous literature (e.g., Carlson and Moses, 2001; Perner et al., 2002a; Devine et al., 2016), results showed a significant relationship between EF and ToM. This relationship was still significant for third-graders in the exploratory correlational analyses split by age group. This result supports the EF-ToM relationship beyond the preschool years, and presumably captures the proposed role of EF in enabling the necessary cognitive processes for performing ToM tasks (e.g., Apperly, 2012).

Results also provided global support for our predictions concerning relationships between cognitive skills and prejudice. First, correlational analyses about individual differences (that is, with the whole sample) showed that both cognitive flexibility and WM were significantly associated with prejudice and counter-bias scores. Interestingly, inhibitory control did not show any significant association with prejudice. Further analyses confirmed that cognitive flexibility was a significant predictor of both prejudice and counter-bias after controlling by composite IQ (and by age when predicting counter-bias). Secondly, correlational analyses for each age group revealed slightly different relationships between cognitive skills and prejudice. For both age groups, significant relationships between EF and ToM emerged; however, only younger children showed a marginal relationship between affective ToM and prejudice. Importantly, regression analyses suggested that different EF processes may underlie prejudice regulation in each age group. In our view, these results shed light on the cognitive factors responsible for prejudice regulation in childhood. Studies on adults’ prejudice regulation mainly highlight the role played by inhibitory control skills. In the present study, inhibitory control processes that are presumably responsible for inhibiting unwanted and non-socially desirable answers seem to be the mechanism underlying prejudice regulation in older children. Conversely, our results provide support for the role of cognitive flexibility on enabling preschool children to engage in a reflective processing of social information, as it had been suggested by Enesco et al. (2009). Whereas inhibitory control seems to operate by facilitating the suppression of automatic stereotypical tendencies in favor of socially accepted, egalitarian behaviors, we presume that cognitive flexibility may facilitate children’s tendency to call stereotypes into question. Moreover, cognitive flexibility skills may underlie a flexible assessment of people according to a variety of dimensions beyond the salient features usually used to categorize people (e.g., skin color). One point of inquiry is that cognitive flexibility’s predictive power seems to be higher in younger children. This could be attributed to the likely limitation of our Dots task when it comes to capture older children’s individual differences in cognitive flexibility skills. Moreover, our results suggest that individual differences in older children’s inhibitory control skills are accounting for children’s ability to suppress their prejudiced attitudes. In other words, since a global decrease of prejudice in middle-aged children was not observed, it is possible that, by middle childhood, individual differences in the ability to regulate explicit prejudice play a key role in inhibiting prejudiced attitudes. However, since analyses on age-related distinctive relationships between EF, ToM and prejudice are exploratory, further studies are needed for a better understanding of the EF role in prejudice. Accordingly, forthcoming research should address whether EF acts more as a regulatory mechanism that prevents children from expressing unwanted prejudiced behaviors or more as a facilitator of a deeper cognitive processing of social categories leading to a reduction in prejudice.

Finally, only the affective ToM component was significantly related to the counter-bias score when analyzing individual differences (and only marginally significant in preschoolers). This result is in accordance with that of Fitzroy and Rutland (2010). In fact, they found that performance on a false-belief task focused on feelings predicted regulation of prejudice. Accordingly, it seems pertinent to disentangle cognitive and affective ToM components, as they distinctively contribute to prejudice. However, affective ToM did not play a predictive role of counter-bias. It could be possible that our second-order false belief task did not fully account for the emotional empathy aspect of affective ToM. Arguably, the task used by Fitzroy and Rutland (2010) involved a character victim of a social transgression, which may trigger more empathic feelings for participants. Consequently, in order to test whether affective ToM predicts prejudice, affective ToM tasks should likely assess reasoning about feelings and trigger empathic concern at the same time. Possibly, an effective affective ToM task would test participants’ ability to reason about others’ feelings in interpersonal situations where the negative feelings experienced by a victim are due to the damage caused by a perpetrator’s intentional behavior.

### Limitations and Future Research

This study analyzed developmental changes between early and middle childhood. Given protracted children’s cognitive development, we expect age-related changes in the late childhood that is out of our scope. Widening the age range of the sample could presumably result in significant developmental changes in prejudice. We explored possible age-related differences in relationships between EF, ToM, and prejudice. Since these results are exploratory, more evidence about whether the relationship between cognitive skills and prejudice changes along the development is needed. Another key aspect to consider is the measure of prejudice. Possibly the MRA is an explicit measure that mainly focuses on stereotyping of Romany people. Accordingly, future studies should consider including implicit measures that allow the assessment of automatic biases. Moreover, given the involvement of motivational factors in resistance to express prejudice (e.g., Devine, 1989; Dunton and Fazio, 1997; Payne, 2005) future studies should include the assessment of motivation to control prejudice as a factor that can independently or jointly with EF make a contribution to avoid the expression of prejudice. Finally, future research should address the possibility that the improvement of cognitive skills could impact on the expression of prejudice. Previous research has shown that cognitive skills can be enhanced through interventions (e.g., Rueda et al., 2005; Bianco et al., 2016). Consequently, it is expected that interventions to improve EF and ToM would potentially impact on prejudice expression.

## Ethics Statement

This study obtained approval from the University of Granada Ethics Committee (approval number 208/CEIH/2016). All participants had informed parental consent.

## Author Contributions

ÁH, MR, and RR-B conceived and designed the study. ÁH collected and analyzed the data. MR and RR-B revised and commented on the manuscript. ÁH wrote the final manuscript.

## Conflict of Interest

The authors declare that the research was conducted in the absence of any commercial or financial relationships that could be construed as a potential conflict of interest.
