# Supplementary material for: Children’s Individual Differences in Executive Function and Theory of Mind in Relation to Prejudice Toward Social Minorities
Source: Front Psychol. 2019 Oct 9;10:2293. doi: 10.3389/fpsyg.2019.02293 (PMC6797813; doi:10.3389/fpsyg.2019.02293)
Supplement: Supplementary file 1 [file Table_1.docx]

Supplementary Material

Children’s individual differences in executive function and theory of mind in relation to prejudice toward social minorities

Ángela Hoyo*, M. Rosario Rueda, Rosa Rodríguez-Bailón

*** Correspondence:** Ángela Hoyo: ahoyo@ugr.es

# Supplementary Data

Second-order false belief stories (Miller, 2013):

Cognitive theory of mind stories

Soccer

1. Ana and Juan are getting ready to go to soccer practice. They have practice every afternoon after school. They’re going to meet at the soccer field with the other players on the team.

2. Ana gets to the soccer field before Juan. When she gets there she sees men working on the field. The coach tells her, “The men are putting in new grass. We can’t practice here today. Instead we’re going to practice at the park.”

3. Ana decides to stop at Juan’s house to tell him that practice will be at the park. But before she gets there Juan gets a phone call. It’s the coach. The coach tells him, “we can’t use the soccer field today – the men are putting in new grass. Soccer practice today will be at the park.” Juan says, “OK, see you there.”

4. When Ana gets to Juan’s house Juan’s mom answers the door. She tells Ana, “Juan’s not here. He went to soccer practice.”

(Turn Picture)

*Now Ana did not see that Juan talked to the coach. She did not see that.*

Comprehension questions:

Reality Question: Where has Juan gone for soccer practice?

Memory Question: Where was the soccer practice before the men came to work?

Theory of mind questions:

Test Question: Where does Ana think Juan has gone?

Forced Choice: Does Ana think Juan has gone to the soccer field or to the park?

Justification: Why does Ana think Juan is there?

Ice Cream

1. Jorge and Alba are together in the park in the morning. Alba would like to buy ice cream from the man selling it there, but she has left her money at home. “Don’t be sad,” says the ice cream man, “you can fetch your money and buy some ice cream later. I’ll be here in the park in the afternoon too.” “Oh good,” Alba says, “I’ll come back this afternoon then.”

2. After Alba has left, Jorge notices the ice cream man leaving the park. “I’m going to drive my van to the church,” the ice cream man tells Jorge, “there is no one in the park to buy ice cream.”

3. As the ice cream man drives over to the church he passes by Alba’s house. Alba is looking out the window and spots the van. “Hello Alba!” the ice cream man waves, “I’m heading over to the church, hopefully I’ll be able to sell more ice cream there.” Alba says, “It’s a good thing I saw you, I’ll meet you there this afternoon.”

4. After lunch Jorge heads over to Alba’s house, but she is not at home. “She’s just left to buy ice cream,” Alba’s mother says.

(Turn Picture)

*Now Jorge did not see that Alba talked to the ice cream man. He did not see that.*

Comprehension questions:

Reality Question: Where has Alba gone to buy her ice cream?

Memory Question: Where was the ice cream van in the morning?

Theory of mind questions:

Test Question: Where does Jorge think Alba has gone?

Forced Choice: Does Jorge think Alba has gone to the park or to the church?

Justification: Why does Jorge think Alba is there?

Affective theory of mind stories

Zoo

1. Antonio and María are in the schoolyard before the bell rings and they are very excited. Today their class is taking a trip to the zoo. Antonio likes to go on class trips a lot, but María is extra happy today because she really likes animals.

2. Then Antonio decides to go inside while María plays a game of catch. While Antonio is inside he talks to his teacher. The teacher tells him that the trip to the zoo has been cancelled because the school bus that was going to take them broke down. Antonio goes to find María so he can tell her the bad news.

3. Then, while the teacher is walking around the schoolyard he runs into María. “Are you coming on the zoo trip with us?” María asks. “Haven’t you heard?” says the teacher, “the zoo trip was cancelled because the school bus broke down.” This news makes María very sad because she was looking forward to the trip.

4. Antonio sees María across the schoolyard. He walks over to talk to her.

(Turn Picture)

*Now Antonio did not see that María talked to the teacher. He did not see that.*

Comprehension questions:

Reality Question: How is María feeling right now?

Memory Question: How was María feeling before she talked to the teacher?

Theory of mind questions:

Test Question: How does Antonio think María is feeling before he finds her?

Forced Choice: Does Antonio think María is feeling happy or sad?

Justification: Why does Antonio think that María is feeling that way?

Vet

1. Andrés goes over to Paula’s house because he has some bad news. He is very upset because his dog Toby is very sick. Andrés loves Toby very much so he is very sad. “I’m sorry your dog is sick,” says Paula. “I will come over later and visit you to see how you’re doing.” “I would like that,” says Andrés. Then Andrés walks home.

2. Later, Paula starts to head to Andrés’s house and on the way she stops into the vet’s office to check on Toby. The vet tells Paula that he has good news: Toby is going to be okay! Paula can’t wait to get to Andrés’s house so she can tell him.

3. Before Paula gets to Andrés’s house his phone rings. It’s Toby’s vet. “Don’t worry, Andrés,” he says, “Toby’s doing much better, he should be well again in no time.” This makes Andrés very happy. Toby’s going to be okay!

4. Paula hurries over to Andrés’s house. She really wants to tell him the news.

(Turn Picture)

*Now Paula did not see that Andrés talked to the vet. She did not see that.*

Comprehension questions:

Reality Question: How does Andrés feel right now?

Memory Question: How was Andrés feeling when he left Paula’s house?

Theory of mind questions:

Test Question: How does Paula think Andrés is feeling as she walks to see him?

Forced Choice: Does Paula think Andrés is feeling sad or happy?

Justification: Why does Paula think Andrés is feeling that way?


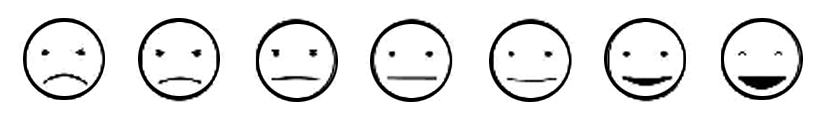


**Supplementary Figure 1.** Scale of faces used in the hidden emotion task. The scale depicted sad (faces 1-3), neutral (face 4) and happy (faces 5-7) emotions. Children received one point if they chose a sad face to describe the character’s emotion.
